# Integrated Pest Management for Stored Grain: Potential Natural Biological Control by a Parasitoid Wasp Community

**DOI:** 10.3390/insects12111038

**Published:** 2021-11-18

**Authors:** Avichai Harush, Elazar Quinn, Anatoly Trostanetsky, Aviv Rapaport, Moshe Kostyukovsky, Daphna Gottlieb

**Affiliations:** 1 Ministry of Agriculture and Rural Development-The Extension Service, Derech HaMaccabim Road 68, P.O. Box 15159, Rishon-LeZion 7528809, Israel; avichaih@shaham.moag.gov.il; 2 Department of Food Science, Institute of Postharvest and Food Science, The Volcani Center, ARO, Derech HaMaccabim Road 68, P.O. Box 15159, Rishon-LeZion 7528809, Israel; elazar@volcani.agri.gov.il (E.Q.); anatoly@volcani.agri.gov.il (A.T.); avivr@volcani.agri.gov.il (A.R.); inspect@volcani.agri.gov.il (M.K.)

**Keywords:** biological control, grain storage, insect pests, IPM, parasitoid wasps

## Abstract

**Simple Summary:**

Parasitoid wasps are well-known biological control agents for arthropod pests in agricultural and forest ecosystems. The stored food product environment is generally also favourable for the parasitoid wasps of the insect pests that infest those food products. Nevertheless, most studies suggest that biological control can reduce pest populations sufficiently only when combined with additional pest-management tools. Combining natural enemies and synthetic chemical pesticides is one of the main challenges in integrated pest management (IPM). We estimated, for the first time, the naturally occurring parasitoid community in grain stores before and after use of synthetic chemical pesticides. There is strong evidence that despite the immediate effect of the pesticides on the parasitoid community, over time, the community can recover. Undoubtedly, a lot of research, particularly of the nature of parasitoid wasps’ recovery in grain-storage facilities, is still required. This will reduce chemical use and implement biological control as a successful and important component of stored-product IPM.

**Abstract:**

Insect contamination of stored grain is a major concern for the grain industry. Phosphine is currently the standard fumigant used to control insect pests in stored grain. However, some species and populations of insects that infest stored grain exhibit resistance to this fumigant and consumers are concerned about pesticide residues. Therefore, alternative methods of effective pest control are needed to partially or completely replace the use of phosphine. There is growing interest in biological control via parasitoid wasps. However, there is evidence that biological control will succeed only if used alongside other pest-management measures. Integrating biological control with the use of chemical insecticide is challenging and may lead to severe reductions in parasitoid survival and success. The main aim of the current study is to shed light on a greatly overlooked issue: the parasitoid community found in stored grain before and after phosphine treatment. The current study results indicate that there is a high level of parasitoid biodiversity within grain stores. We found common parasitoids at both semi-arid and Mediterranean sites, suggesting that those parasitoids can be active across a wide range of abiotic conditions. This research indicates that the community may recover even though phosphine has an immediate negative effect on a parasitoid community. Nevertheless, the parasitoid wasps seem to reduce the host population insufficiently. In light of the findings presented here, those interested in implementing pest-management strategies that include both phosphine treatment and biological control should consider conservation and augmentation of the naturally occurring parasitoid population. These studies should take into account interactions between and within parasitoid populations and phosphine distribution within the grain storage. To limit the effect of phosphine on the parasitoids, pest-management strategies should also reflect careful consideration of the timing of phosphine treatment and the need for sufficient refuge for the parasitoids.

## 1. Introduction

Among insects, some species can cause major economic losses in stored grain and other stored commodities. They can threaten food security worldwide and damage other products, including fiber, leather and wood [1]. The environmental conditions inside storage structures provide an excellent environment for rapid and successful development of insect populations, which can lead to severe damage and economic losses. Phosphine is the standard fumigant used to control pests in stored grain around the world; however, many species and populations of stored-product insects are showing resistance to this fumigant [2]. In addition, phosphine is corrosive and can damage equipment if used frequently and at high concentrations [2]. These issues and the growing consumer demand for foods free of chemical additives, toxins and pesticide residues provide strong reasons to search for alternative tactics for controlling insects in stored grain. 

Interest in parasitoid-based biological control of pests of stored products has grown considerably in recent decades and has mostly focused on Bethilidae, Braconidae, Ichneumonidae and Pteromalidae parasitoids [3,4]. The stored food product environment is generally favorable for the natural enemies of the insect pests that infest those food products. Nevertheless, most studies suggest that biological control can reduce pest populations sufficiently only when combined with additional pest-management tools [5]. Combining natural enemies and synthetic chemical pesticides is one of the main challenges in integrated pest management (IPM). The primary challenge is that parasitoids can be just as or more susceptible to chemical insecticides than their hosts [6]. For this reason, the use of natural enemies against stored-product insects has been largely limited to organic, small-scale farms. Only a limited number of inorganic companies such as tobacco, pasta and chocolate companies, see review [7,8] practice IPM with parasitoid wasps (e.g., Trichogrammatidae: *Trichogramma evanescens* [Westwood] Braconidae: *Habrobracon hebetor* [Say], *Cephalonomia tarsalis* [Ashmead] and Pteromalidae: *Anisopteromalus calandrae* [Howard]).

Studying the parasitoid populations in stored grain before and after phosphine treatment may not only suggest the potential of IPM, but also assist the design of pest-management strategies focused on conservation of the local parasitoid species. Conservation and augmentation of the native parasitoid community is always preferable to the use of imported exotic species, reviewed in [8]. Any introduction of exotic parasitoids to stored grain can lead to competitive interaction between parasitoids, reducing their pest-control effect. There is also a risk of the death of the exotic species due to poor acclimation to the new environment, reviewed in [8]. Thus, the first step toward the integration of biological control in non-organic grain storage systems is the identification and cataloging of the native parasitoid community [9]. Surveys of naturally occurring parasitoid community in stored grain have been conducted in Pakistan, five species [10], Egypt, 16 species [11], the USA, eight species [12], Greece, 16 species [7], Iran, 10 species [13], and Sicily, five species [3]. These surveys and others suggest that stored grain can sustain a stable community of parasitoids. In the current study, we evaluated the effect of phosphine treatment on the structure of the parasitoid community in stored wheat. Insufficient monitoring of phosphine concentration during fumigation and inadequate sealing of storage facilities, are common and known to lead to quick recovery of insect pests in the storage [14]. Thus, we predicted that (1) parasitoid abundance and the composition of the parasitoid community would be strongly affected by the phosphine treatment, however (2) recovery of the parasitoids may occur due to phosphine unequal distribution.

## 2. Materials and Methods

### 2.1. Study Area

The study was conducted at two sites in Israel: a site located in the Jezreel Valley in the Mediterranean climate zone (henceforth the Med. site) and a site located in the semi-arid western Negev desert (henceforth the Ar. site). At each site, there were two separate grain storehouses. Each storehouse had a metal roof, with an opening for ventilation running around the structure just below the roof. All storehouses were 2000 m^2^ in area (10 m height, 25 m × 80 m floor dimensions) and held 12,000–2000 tons of mainly local wheat. The average distance between the storehouses at each site was 150 m and all of the storehouses had similar spatial features and directionality. Therefore, we did not expect to find much variation between the storehouses at each site [15]

The wheat was introduced to the storehouse during June–July, 2019. Once a year, the whole pile of grain was fumigated with phosphine. The Med. storehouses were fumigated in October–November (the fourth month of storage) and the Ar. storehouses were fumigated in January–February 2020 (the seventh month of storage). Because the hazardous nature of chemical used for fumigation proper barrier material were used to avoid leakage of phosphine. Two weeks after fumigation, the barrier was removed and there were no phosphine residuals (Personal observation by EQ, AT and MK). A year after the introduction of the wheat to the storehouse, the storehouse was completely emptied and treated with insecticides before being filled with new grain.

Since grain was gradually removed from the storehouse, we examined only storehouses that held between 3000 and 12,000 tons of mainly local wheat. The storehouses at the two sites were managed by the same technicians using the same fumigation and sampling procedures.

### 2.2. Survey

Eight samples from the center and the margins of each storehouse, with a distance of at least 10 m between sampling points in each storehouse, were collected monthly, to assess the temperature between the grains of wheat, grain moisture content and the presence of insect species.

The temperature between the grains was estimated using a thermocouple probe that was pushed into the grain bulk. Based on previous reports [15] of a strong correlation between the relative ambient humidity and grain water content, all of the statistical analyses in this study included only grain water content. Grain moisture content was estimated in the lab using an Accurate Multilingual Moisture Tester (GAC 2100, DICKEY-john, Road Auburn, IL 62615, USA).

The presence of live insects was directly tested in 1 kg of grain collected with a re-sealable plastic bag (common method for monitoring pests [15] and parasitoids [16]) from the center and margins of the grain bulk at both sites. The samples were collected from 0 to 40 cm below the grain surface. Since we collected only at that depth, we assume that species that exploit deeper areas within the grain bulk, e.g., the parasitoid *Lariophagus distinguendus* [17] and the pest *Rhyzopertha dominica* will not be fully represented in our samples. Insect samples were preserved in 70% ethanol at 4 °C. All arthropods were later identified to at least the order level. Parasitoid wasps were identified to the genus or species level, with the help of keys [18,19,20] and consulting experts.

### 2.3. Data Analysis

Samples that were taken at each site on each date were considered to be pseudo-replications. In all of the statistical tests performed as part of this study, the storehouse was defined as a random factor. The variance in the biotic and abiotic factors between the storehouses accounted for 0.025–0.042% and 0.046–0.067% of the total variance at the Mediterranean and semi-arid sites, respectively. Thus, all graphs present the combined data from both storehouses at each site.

We first calculated the average number of parasitoids in each group, for the abundance analyses. To categorize the abundance of each species, the criteria of *frequency* and *dominance* were used, as suggested by Athanassiou and Buchelos (2001). A species’ frequency is the percentage of samples in which it was detected. A species can be categorized as *constant* (>50%), *accessory* (25–50%), or *accidental* (<25%). Dominance refers to the percentage of collected individuals that are members of a given species. A species can be classified as *dominant* (>5%), this value seems low but is widely accepted and referred to in the study of grain storage pests.

We tested whether the abundance of each pest (i.e., host) species was affected by the abundance of their parasitoid wasps or the examined abiotic factors (i.e., Ar. Site vs. Med. site, center vs. margin of the grain bulk, grain moisture content and grain temperature). We used zero-inflated generalized linear mixed models with a negative binomial distribution and log-link function. This type of model was chosen because many of the samples contained no parasitoids and the data distribution deviated from Poisson’s distribution. In addition, we compared the effect of phosphine fumigation (i.e., the difference between species abundance a month before fumigation and after fumigation) at both sites, by estimating species composition turnover between the two time points.

Statistics were performed using the R Core Team (2017). We used ‘lme4’ [21], ‘lmerTest’ [22] and ‘MuMIn’ [23] packages for GLM, ‘relaimpo’ [24] and ‘codyn’ packages [25] to analyze species turnover in community similarity between two time points.

## 3. Results

### 3.1. Parasitoid Distribution by Family

Overall, 243 individual parasitoid wasps were sampled, representing four families: Pteromalidae, Bethylidae, Braconidae and Chalcididae. All of the parasitoid wasps that we found are known to be idiobiont ectoparasitoids, mainly attacking host’s larvae and pupae developmental stages. The parasitoids’ potential hosts are summarized in Table 1.

### 3.2. Parasitoid Dominancy and Abundance

**Figure 1:**
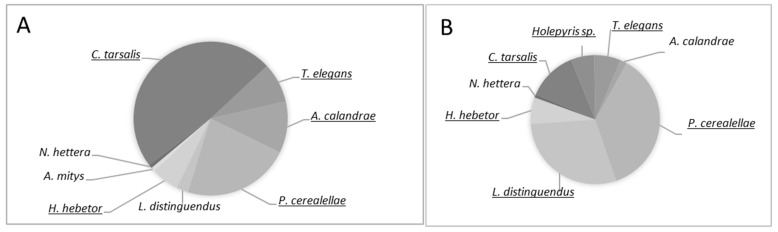
Species dominance at the (**A**) Med. and (**B**) Ar. sites. The names of dominant species are underlined.

During the whole study period, there were a total of 122 individual parasitoid wasps at the Med. and 121 individual parasitoid wasps at the Ar. site. At both sites, the frequency of all species was found to be *accidental* (following categories detailed in Methods); four species, at both sites, were found to be *dominant* (Latin name underlined in Figure 1). *Anisopteromalus calandrae* was only *dominant* at the Med. site, *Holepyris* sp. was only found at the Ar. site and *Antrocephalus mitys* was found only at the Med. site (Figure 1).

### 3.3. Parasitoids Affect the Abundance of Pest Species

The main pest species found in the storehouses were species that are common in grain-storage facilities in Israel: the saw-toothed grain beetle, *Oryzaephilus surinamensis* (L.); the lesser grain borer, *Rhyzopertha dominica* (F.); the rice weevil, *Sitophilus oryzae* (L.) and the red flour beetle, *Tribolium castaneum* (Herbst). Lepidoptera larvae found in the 1-kg grain sample were potentially the Indian-meal moth, *Plodia interpunctella* (Hübner) and the Angoumois grain moth, *Sitotroga cerealella* (Olivier).

All hosts were significantly affected by temperature, grain moisture and one parasitoid wasp (Table 2), except for *S. oryzae*, which was significantly affected by five parasitoid wasps. *Holepyris* sp. had no significant effect on any of the hosts. Both *A. calandrae* and *C. tarsalis* affected more than one host. Except for *T. castaneum* and *R. dominica* all hosts were significantly more abundant at the Med. site and within the storehouses, all of the species, except for the moths and *R. dominica*, were significantly more abundant at the storehouse margins (*p* < 0.0001). *Rhyzopertha dominica* did not show any significant preference for location within the storehouse (*p* > 0.05) and the moth significantly preferred the margins of the storehouse (*p* < 0.0001).

### 3.4. Effect of Phosphine Treatment on the Composition of the Parasitoid and Pest Community

**Figure 2:**
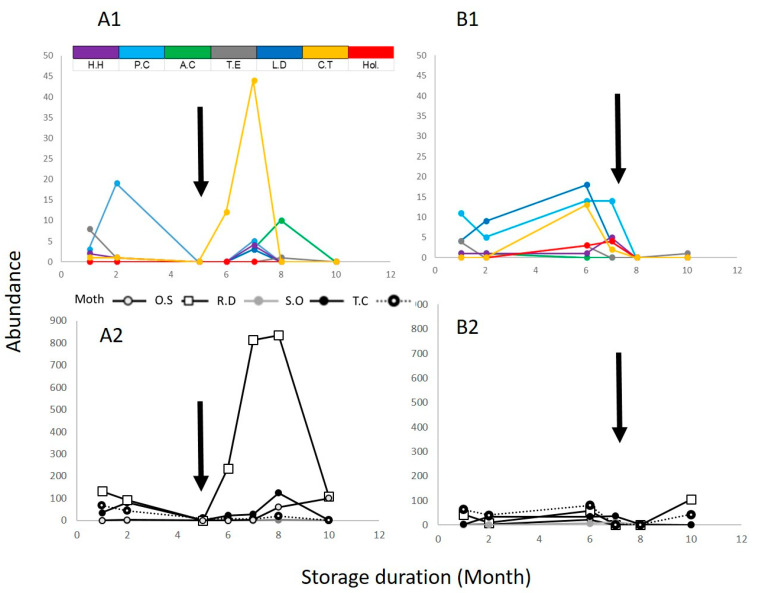
Abundance of (**1**) parasitoid wasps and (**2**) insect pests at (**A**) the Med. site and (**B**) the Ar. site. Arrows indicate time of exposure to phosphine. OS—*Oryzaephilus surinamensis*; SO—*Sitophilus oryzae*; TC—*Tribolium castaneum*; RD—*Rhyzopertha dominica*; Moth—*Plodia interpunctella* and *Ephestia cautella.* Wasps: CT—*Cephalonomia tarsalis*, *Holepyris* sp., HH—*Habrobracon hebetor*, LD—*Lariophagus distinguendus*, PC—*Pteromalus cerealellae*, TE—*Theocolax elegans*, AC—*Anisopteromalus calandrae*.

The abundance of both wasps and hosts was significantly reduced immediately after the phosphine treatment (Figure 2). At the following months, the host and wasp populations reappeared. At the Med. site, all parasitoids before phosphine treatment appeared after phosphine treatment with two additional species: *L. distinguendus* and *A. calandrae* (community turnover rate of 0.21 with 0.21 appearance rate). All hosts before phosphine treatment appeared after phosphine treatment accept for *R. dominica* (community turnover rate of 0.20). At the Ar. site, most parasitoids before phosphine treatment disappeared after phosphine treatment with the reappearance of only one species: *T. elegans* (community turnover rate of 1.00 with 0.89 disappearance rate). All hosts before phosphine treatment appeared after phosphine accept for *R. dominica*, *S. oryzae* and moths (community turnover rate of 0.40).

## 4. Discussion

Combining natural enemies and synthetic chemical pesticides is one of the main challenges in IPM [6]. There have been several cases in which parasitoids have been found to be just as or more susceptible to chemical insecticides than their respective hosts [6]. The current study provides strong evidence that, despite the use of phosphine there is high parasitoid diversity suggesting potential future development of IPM.

The identification of the naturally occurring parasitoids and their hosts is the first step toward understanding the underlying host–parasitoid interaction in stored grain. The relative abundance of the different host species at each site was congruent with the findings of a previous 10-year study of host abundance at those sites [15]. In both studies, the most abundant pest species were *O. surinamensis*, *T.* *castaneum* and *S.* *oryzae* with the community recurring after phosphine treatment. Furthermore, as in the current study, in the Med. site *O. surinamensis* is throughout the year the most abundant pest species while at the Ar. site the most abundant species switches throughout the year. Thus, we presume that the current one-year study is a good example of the community dynamics at the two sites. The most abundant parasitoids in the grain samples were the beetle parasitoids *C. tarsalis*, *Holepyris* sp. (only at the Ar. site), *L. distinguendus*, *P. cereallae*, *T. elegans* and *A. calandrae* (only at the Med. site). At both sites, these species represented more than 90% of the adult wasps (Figure 1). These species are commonly found in stored grain around the world and are suggested as good candidates for biological control, e.g., [10,11,12]. At both sites, we observed a strong association between a host and at least one of the parasitoids (Table 2). Nevertheless, the fact that there was a greater abundance of host insects at the Med. site, but similar abundance of parasitoid wasps at both sites, suggests that the parasitoids did not provide very good pest control. Intriguingly, except for *S. oryzae*, the abundance of each host species correlated to only one parasitoid. Similarly, although more than 70% of the parasitoids at the Med. site and 40% at the Ar. site are known to parasitize moth larvae, *H. hebetor* was the only wasp found to be associated with moths. The almost unique association with a host of both beetle and moth parasitoids suggests that community structure and host–parasitoid interactions are probably influenced by intraspecific and interspecific competition. Competition among parasitoids can influence the size, structure and stability of insect communities, all of which are important for biological control [32]. Following this study, detailed study on the competitive interactions, within and between the “dominant” parasitoid, underlying these correlations’ will increase IPM efficiency and especially the practice of parasitoid augmentation.

It seems that phosphine’s immediate role is in reducing the community abundance. The parasitoids’ successive recovery can be an outcome of species becoming resistant to phosphine, reviewed in [9], but see also [33]. However, we assume that most of the parasitoids found in the stored grain did not develop resistance and the recovery is probably an outcome of phosphine heterogenic distribution. Heterogenic distribution may occur by two non-mutually exclusive conditions: low level of diffusion of phosphine into grain kernels [33] and the presence of grain that had not been reached by the phosphine treatment. These conditions are a common concern in terms of pest management but can be beneficial for the parasitoid community.

The penetration and diffusion of phosphine into grain kernels are limited, i.e., pest species that develop outside grain kernels are usually more susceptible to fumigants than pest species that develop inside grain kernels [33]. Correspondingly, parasitoids that oviposit within grain kernels (*L. distinguendus*, *P. cerealellae*, *T. elegans*, *A. calandrae*) are less exposed to phosphine than parasitoids of external grain consumers (*C. tarsalis*, *Holepyris* sp., *H. hebetor*). At the Ar. site it might seem that diffusion plays a role, i.e., only *T. elegans* reoccurs. But, at the Med. site all parasitoid species reoccurred. This suggests that species reoccurrence after phosphine treatment is most probably not an outcome of phosphine limited diffusion into the grain.

**Figure 3:**
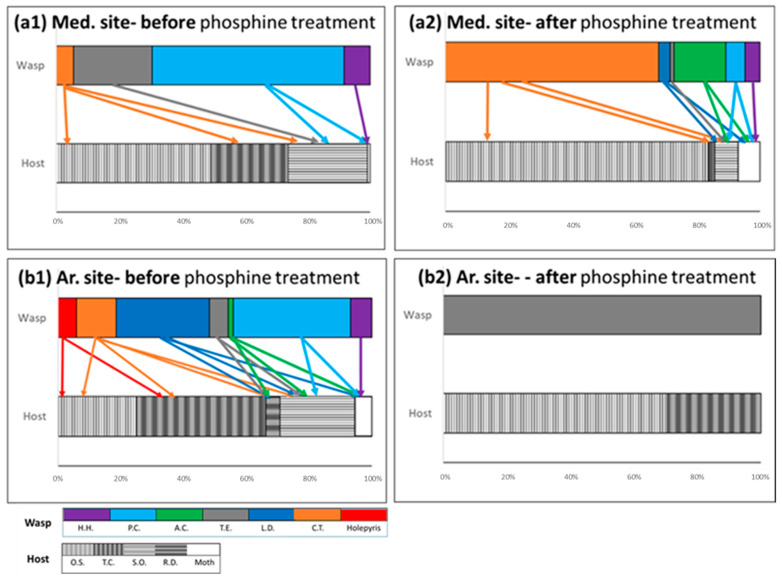
Wasp and host abundance (%) at the (**a**) Med. site and the (**b**) Ar. site (**1**) before and (**2**) after the application of phosphine. Arrows indicate on parasitoid wasp- host interaction based on Table 1. OS—*Oryzaephilus surinamensis*; SO—*Sitophilus oryzae*; TC—*Tribolium castaneum*; RD—*Rhyzopertha dominica*; Moths: *Plodia interpunctella* and *Ephestia cautella.* Wasps: CT—*Cephalonomia tarsalis,* Hol.—Holepyris sp., HH—*Habrobracon hebetor*, LD—*Lariophagus distinguendus*, PC—*Pteromalus cerealellae*, TE—*Theocolax elegans*, AC—*Anisopteromalus calandrae*.

The low level of variability between the storehouses at each site is probably due to insects moving between the structures. This may strengthen the idea that areas at the grain-storage site that are not exposed to phosphine treatment might be the main explanation for the relatively rapid appearance of parasitoids and their hosts after phosphine treatment. Those areas such as cracks in the wall, crevices, aeration ducts or any other area that is difficult to clean and does not get phosphine treatment or any nearby-untreated storage can provide parasitoids and their hosts with a refuge from the phosphine treatment. For example, Bethylids are known for their ability to access these critical refuge environments [34]. Mapping phosphine distribution and refuge was not in the scope of the current study. Future studies should incorporate this element for improved use of phosphine on pests, on the one hand and improving parasitoid abundance on the other. Different undetected possibility of refuges between the Med. and Ar. sites may explain the different recovery patterns of the parasitoids. At the Ar. site, the high turnover of the parasitoid community structure indicates the limited ability of the refuge to preserve the parasitoid community. Recovery may also depend on the different timing of phosphine treatment at the two sites. The abundance of hosts and their associated parasitoids recovered significantly, after phosphine treatment, at the Med. site; whereas at the Ar. site, recovery was small. The tradeoff between phosphine treatment early in the season to maintain a lower turnover rate of parasitoids (e.g., at the Med. site) and late phosphine treatment to avoid greater pest population growth before grains are removed (e.g., at the Ar. site) should be taken into account when planning a sustainable IPM program. Furthermore, to increase effective control of parasitoids, augmentation of the naturally occurring parasitoids that are strongly associated with the pests and do not compete with each other should be accounted for. Based on the current study, *H. hebetor*, *C. tarsalis*, and *A. calandrae* cover all existing main pests with high associations. In the Med. site recurrence of all three parasitoids after phosphine, suggest that augmentation can occur before phosphine treatment (Figure 3(a1,2)). However, in the Ar. site (Figure 3(b1,2)) none of at the three species reoccurred after phosphine treatment. Thus, it seems that it is essential to conduct augmentation at the Ar. site before and after phosphine treatment of only *C. tarsalis* since the main pests are *O. sitophilus* and *T. castaneum.*

## 5. Conclusions

This study provides the first reported data regarding the presence of native parasitoid wasps in stored grain before and after phosphine treatment. There is strong evidence that despite the immediate effect of phosphine on the parasitoid community, the community can recover over time. Currently, the parasitoid wasps do not seem to sufficiently reduce pest populations, but do show strong association with the host abundance. Thus, future studies should consider supplying refuge habitat for those wasps, as well as conservation and possible augmentation of the naturally occurring parasitoid populations. The current study suggests that augmentation of three native “dominant” parasitoid species can increase efficacy pest management. There is a great deal of literature on the nature of the interactions between these species in grain-storage facilities [3,4,5,6,7,8,9]. This study is the basis for understanding the interactions pre- and post phosphine fumigation. Additional future studies are required in order to reduce chemical use and implement biological control as a successful and important component of stored-product IPM.

## Figures and Tables

**Table 1:** List of main insect pests in the stored grain and their associated parasitoids.

Species in the Grain Storehouses		Curculionidae	Bostrichidae	Tenebrionidae	Silvanidae	Moth Larvae
		*Sitophilus oryzae* (Linnaeus)	*Rhyzopertha dominica* (Fabricius)	*Tribolium* sp. (Macleay)	*Oryzophilus surinamensis* (Linnaeus)	
Bethylidae	*Cephalonomia tarsalis* (Ashmead)	[26]	[26]	[26]	[26,27]	
	*Holepyris* sp.			[26]	[26]	
Braconidae	*Habrobracon hebetor* (Say)					[26]
Chalcididae	*Antrocephalus mitys* (Walker)					[28]
	*Neohybothorax hettera* (Walker)	Unknown [29]	Unknown [29]	Unknown [29]	Unknown [29]	Unknown [29]
Pteromalidae	*Lariophagus distinguendus* (Förster)	[26,30]	[30]			[26]
	*Pteromalus cerealellae* (Ashmead)	[31]				[26]
	*Theocolax elegans* (Westwood)	[26,30]	[26,30]			[26]
	*Anisopteromalus calandrae* (Howard)	[26,30]	[26,30]			[26]

**Table 2:** The effect of the abundance of dominant parasitoid-wasp species on the abundance of their hosts.

Pest (Host)	Abiotic Conditions				Parasitoid Wasps						
	Location in Storehouse	Temperature	Grain Moisture	Site	*Holepyris* sp.	*C. tarsalis*	*L. distinguendus*	*T. elegans*	*A. calandrae*	*P. cerealellae*	*H. hebetor*
*O. surinamensis*	<0.0001	<0.0001	<0.0001	<0.0001	NS	<0.0001					
*T. castaneum*	<0.0001	<0.0001	<0.0001	NS	NS	<0.05 *					
*R. dominica*	NS	<0.05	<0.001	<0.01		NS	NS	NS	<0.01		
*S. oryzae*	<0.0001	<0.0001	<0.0001	<0.0001		<0.0001	<0.05	<0.01	<0.0001	<0.01	
Moth	<0.0001	<0.0001	<0.0001	<0.0001	NS		NS	NS	NS	NS	<0.0001

* Interaction between the parasitoid wasp and site. NS—not significant; *p* > 0.05.

## Data Availability

Not applicable.
